# Syntenin overexpression in human lung cancer tissue and serum is associated with poor prognosis

**DOI:** 10.1186/s12885-020-6653-6

**Published:** 2020-02-27

**Authors:** Pengyong Luo, Xuli Yang, Shiren Huang, Shu Feng, Zongxing Ou

**Affiliations:** Department of Respiratory Medicine, Haikou Hospital Affiliated to Xiangya Medical College, Central South University, 43 People’s Avenue, Haikou, 570208 Hainan China

**Keywords:** Syntenin, VEGF, Lung cancer, Expression, Prognosis

## Abstract

**Background:**

Lung cancer is the major malignant tumour. The present study was conducted to determine the expression level of syntenin in lung cancer tissues and serum from lung cancer patients and to explore its clinical significance.

**Methods:**

Syntenin expression levels were determined in paraffin-embedded lung cancer tissue specimens (*n* = 191) using immunohistochemistry. The mRNA expressions of syntenin in fresh lung cancer tissues and the paracancerous tissues were examined by RT-qPCR (*n* = 25). Syntenin and VEGF expression levels were measured in serum from patients with lung cancer (*n* = 60) and control subjects (*n* = 30) using ELISA. The associations between syntenin and the clinicopathological features or prognosis in 191 patients with lung cancer were analysed. The correlation between the syntenin and VEGF levels in serum from 60 lung cancer patients was analysed.

**Results:**

The expression levels of syntenin were significantly higher in lung cancer tissues than in paracancerous tissues based on immunohistochemistry and RT-qPCR, and elevated syntenin expression was significantly associated with tumour size (*P* = 0.002), TNM stage (*P* = 0.020), tumour distant metastasis (*P* = 0.033), overall survival (OS) (*P* = 0.002) and progression-free survival (PFS) (*P* = 0.001). Multivariate analysis revealed that increased expression of syntenin was an independent risk factor for OS (*P* = 0.006) and PFS (*P* < 0.001) in lung cancer patients. The expression levels of syntenin and VEGF in serum from lung cancer patients were higher than those from control subjects (*P* < 0.001, *P* < 0.001, respectively), and their expression levels were positively correlated (*r* = 0.49, *P* < 0.001).

**Conclusions:**

Syntenin expression is upregulated in lung cancer patients, and its serum expression level is positively correlated with VEGF. Moreover, syntenin overexpression was correlated with poor prognosis in patients with lung cancer.

## Background

The global morbidity and mortality due to malignant lung cancer is ranked the first [1], which seriously threatens human health and safety. Tumour invasion and metastasis are considered to be the main causes of treatment failure [2, 3]. At present, lung cancer is mainly treated with surgery supplemented with radiotherapy, chemotherapy, targeted therapy and immunotherapy. However, due to metastasis and drug resistance, the treatment efficacy is inadequate, new therapeutic targets and improved treatment strategies are being sought continuously.

Although targeted molecular drugs improve the therapeutic effect of lung cancer, they are largely limited to patients with specific gene mutations. Angiogenesis is the formation of new blood vessels from the existing vascular network, which is more attractive and common in the development of targeted therapy [4]. In the first-line and second-line treatments, VEGF bevacizumab or VEGF receptor (VEGFR) ramolumab alone or in combination with chemotherapy prolong the total survival period [5]. While targeted management of angiogenesis is effective, there are too many potential signal pathways for angiogenesis, and the high heterogeneity of tumor may cause difficulties in clinical benefits of anti angiogenic therapy [6, 7]. Furthermore, at present, there are only few targeted drug for angiogenesis. At the same time, the resistance of drug targeting anti-angiogenesis is a serious issue, and it is necessary to find new therapeutic targets.

Syntenin, the melanoma differentiation-associated gene-9 (MDA-9), is involved in multiple signal cascades in the physiological and pathological processes of cells [8–10]. The roles of syntenin in tumour migration and invasion have been intensively investigated. The results showed that Syntenin binds to kinases or receptors on the cytoplasmic membrane to activate the P38/NF-kB, ERK1/2 pathways, AKT pathways, P38/MAPK, leading to progression and metastasis in various malignant tumours, such as melanoma, glioma, breast cancer, small cell lung cancer and liver cancer [11–15]. It is shown that the up-regulation of syntenin promotes the migration of non-metastatic cancer cells [16], and knockdown of syntenin inhibits the migration and invasion of cells [17, 18]. In addition, syntenin has been shown to promote the formation of blood vessels [19]. VEGF has been demonstrated to be an important physiological and pathological factor for angiogenesis and play an important role in the occurrence and development of tumor [20]. By binding with VEGFR, it activates the tyrosinase activity of VEGFR, leading to proliferation of vascular endothelium and angiogenesis via the signal transduction [21]. Therefore, syntenin plays a vital role in the metastasis and progression of tumour.

At present, the roles of syntenin in lung cancer are still largely unclear, and there are few reports on the expression of syntenin in lung cancer tissues and its clinical significance. Therefore, the present study was performed to explore the associations between the expression of syntenin in patients with lung cancer and their clinicopathological characteristics and prognosis, and to analyze the expression level of serum syntenin and its correlation with VEGF expression level in patients with lung cancer. The findings would lay the foundation for further investigation of new diagnostic and prognostic markers of lung cancer and new therapeutic targets.

## Methods

### Patients, tissue and serum sample

From the database of patients who were diagnosed with primary lung cancer by pathological examination at Haikou Hospital between 2012 and 2014, 191 lung cancer patients were included with sample size determined based on previous described method [22], including 140 men and 51 women who were aged from 27 to 89 years old. Paraffin tissues from the 191 lung cancer patients and 80 samples of lung tissue adjacent to tumours from the matched group were obtained from the pathology department of Haikou Hospital. Eighty samples of paraffin-embedded lung tissues were obtained from 80 cases of surgical treatment among the 191 patients. These samples were used for determining the expression of syntenin in the lung cancer and paracancerous normal tissues. Patients for immunohistochemistry study were included if their lung cancer was confirmed by pathological biopsy with complete clinical data complete clinical data on gender, age, tumor size, lymph node metastasis and distant metastasis, tumor pathological data, performance status score and treatments. They were followed-up till death of the end of this study with minimal survival time of 1 month. No patient was lost during the study. From June 2019 to August 2019, 25 cases of lung cancer patients with fresh lung cancer tissues and their paracancerous normal lung tissues were used for qPCR analysis. The paracancerous tissue adjacent to cancer was more than 3 cm from the edge of cancer, and all the samples were confirmed by pathological examinations.

The venous blood was extracted from sixty lung cancer patients for analysing serum syntenin and VEGF levels. These patients were diagnosed by pathological biopsy for the first time in the Department of Respiratory Medicine at Haikou Hospital between October 2018 and April 2019, including 41 men and 19 women aged 43 to 79 years old and not subject to anticancer treatment before diagnosis. Thirty volunteers who received physical examinations at the Haikou Hospital were enrolled as the control group. Venous blood was collected in the morning, and the serum was separated after centrifugation and stored at − 20 °C. The lung cancer group and the control group had no significant differences in age, gender and living area.

The exclusion criteria included the presence of other serious acute and chronic diseases, such as severe COPD, cerebral haemorrhage, cerebral infarction, myocardial infarction, hypertension, diabetes, liver cirrhosis, uraemia, sepsis, MODS or combined tumour type, or acute trauma.

### Treatment and follow-up

Among the 191 patients included, 80 received surgical treatment, 76 of them were at stage I, II and resectable stage IIIA, and they underwent segmental or lobectomy plus lymphadenectomy, and 4 of them received palliative lobectomy and other systemic treatment. 111 cases were treated with individualized conservative chemotherapy based on the tumor stage, PS (Performance status) score and patients’ economic situation. 34 of them received EGFR TKI inhibitor gefitinib as the first-line treatment, the rest received platinum chemotherapy and palliative symptomatic supportive treatment. All patients were followed up every 3 month for one to 84 months. The OS was calculated from the diagnosis of lung cancer to the end of follow-up or the patient’s death, and the PFS was calculated from when the patient first received treatment until the first disease progression or the patient’s death. After treatment, patients were evaluated for progress of tumor with chest CT, head MRI, abdomen CT and whole body bone scan.

### Immunohistochemistry

Tissue sections (3 μm) were routinely dewaxed, hydrated and heated to retrieve the antigens. Endogenous peroxidase activity was suppressed with 3% H_2_O_2_ for 20 min at 25 °C. To suppress nonspecific binding of antibody, the tissue sections were immersed in 10% goat serum for 20 min. The slides were incubated with a monoclonal antibody against syntenin (Abcam 133,267, Cambridge, UK, dilution 1:150) overnight at 4 °C. After intensive rinsing in PBS, the slides were incubated with biotin-labelled secondary antibody (MXB Biotechnology, Fuzhou, China) for 15 min at 25 °C. The staining was developed with diaminobenzidine (MXB Biotechnology, Fuzhou, China), and counterstained with haematoxylin, dehydrated and mounted for microscopy. The primary antibody was omitted in the negative control.

The stained sections were reviewed independently by two pathologists who were blinded to the clinical outcomes, and 10 high-magnification microscopic fields were randomly selected for each treatment. The immunoreactive score (IR) [23] was used to calculate immunoreactivity and was calculated as IRS = SI (staining intensity) *PP (percentage of highly-expressing cells). The SI was defined as follows: 0, negative; 1, low; 2, medium; 3, high. The PP was scored as follows: 0, 0–5% stained; 1, 6–25% stained; 2, 26–50% stained; 3, 51–75% stained; 4, 76–100% stained. Finally, the IRS was defined as follows: 0–4; low expression; 5–12, high expression [24].

### Quantitative real-time PCR(RT-qPCR)

Total RNA was extracted from fresh frozen tissue by using TRIzol reagent (Vazyme R401–01-AA, Nanjing, China), and reverse transcriptions were carried out with a PrimeScript RT reagent Kit (Vazyme R223–01, Nanjing, China). Gene-specific primers of human syntenin and β-actin were synthesized at Sangon Biotech (Shanghai) Co., Ltd. The primer sequences were as follows: human syntenin (forward primer, 5′- TTCTGCTCCTATCCCTCACG − 3′ and reverse primer, 5′- CCAGTTACAGGAGCCACCAT − 3′), β-actin (forward primer, 5′- ATCATGTTTGAGACCTTCAACACCCCAGCC − 3′ and 5′- AAGAGAGCCTCGGGGCATCGGAACCGCTCA − 3′). The iCycler and iQ real-time PCR instruments (LighCycler 480II Roche Group Switzerland) and SYBR-Green PCR Master Mix (Vazyme Q711–00, Nanjing, China) were used qRT-PCR. The qRT-PCR conditions were as follows: 95 °C for 5 min; and 95 °C for 30 s, 60 °C for 30 s, and 72 °C for 30 s for 39 additional cycles. All experiments were repeated three times, and the mRNA levels were normalized to that of β-actin as an internal control.

### Enzyme-linked immunosorbent assay (ELISA)

ELISA was conducted according to the instructions in the ELISA kit (Cloud-Clone, Houston, USA). 100 μL of serum or standard product was added to the microplate and incubated at 37 °C for 1 h. The liquid was discarded, and the biotinylated antibody was added and incubated at 37 °C for 1 h. After washing, HRP-labelled antibody was added and incubated at 37 °C for 30 min. After thorough washing, the TMB substrates were added for 20 min at 37 °C in the dark, and dilute sulfuric acid was added to terminate the reaction. The absorbance (A) value was measured at a wavelength of 450 nm on an ELISA instrument (Bio-Rad, California, USA). All samples were repeated three times, and the investigators were blinded to the clinical information.

### Statistical analysis

Statistical analyses were performed using SPSS 23.0 software (SPSS Statistics, IBM, USA). The TNM stage of lung cancer was determined based on the eighth international lung cancer staging standards developed by the International Association for the Study of Lung Cancer. Kaplan-Meier analysis was applied to analyse the survival and prognosis of patient, and univariate and multivariate Cox proportional hazard regression analyses were used for survival-related variable analysis. The Wilcoxon signed-rank test was applied to examine the significance of differences in syntenin mRNA expression between the lung cancer tissue and paracancerous tissue. Spearman correlation analysis was applied to analyse the correlation between the expression of syntenin and VEGF in serum from patients with lung cancer. The expression of syntenin and VEGF in serum was represented as the median (quartile range) [M(P25~P75)]. The Mann-Whitney U test was used to compare continuous variables, and Pearson chi square test was used to compare categorical variables. A value of *P <* 0.05 was considered statistically significant.

## Results

### Syntenin was expressed in lung cancer tissues

**Fig. 1:**
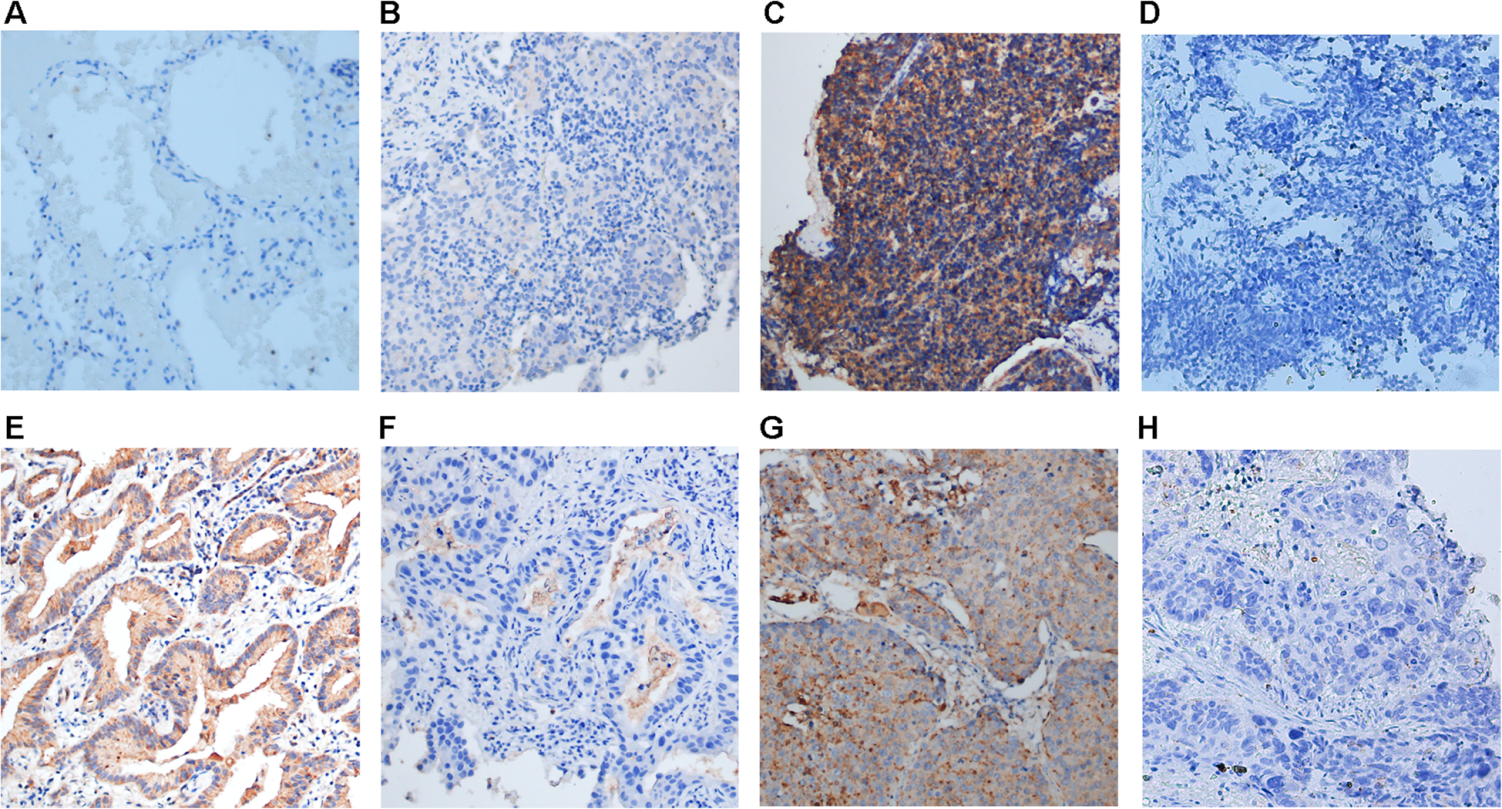
Syntenin expression in lung cancer tissue and lung tissue as detected by immunohistochemistry. The low expression of syntenin in lung tissue (**a**) and large cell lung cancer (**b**); the high and low expression of syntenin in small cell lung cancer (**c**, **d**). The high and low expression of syntenin in adenocarcinoma (**e**, **f**); the high and low expression of syntenin in squamous cell carcinoma (**g**, **h**). Original magnification, × 200

**Fig. 2:**
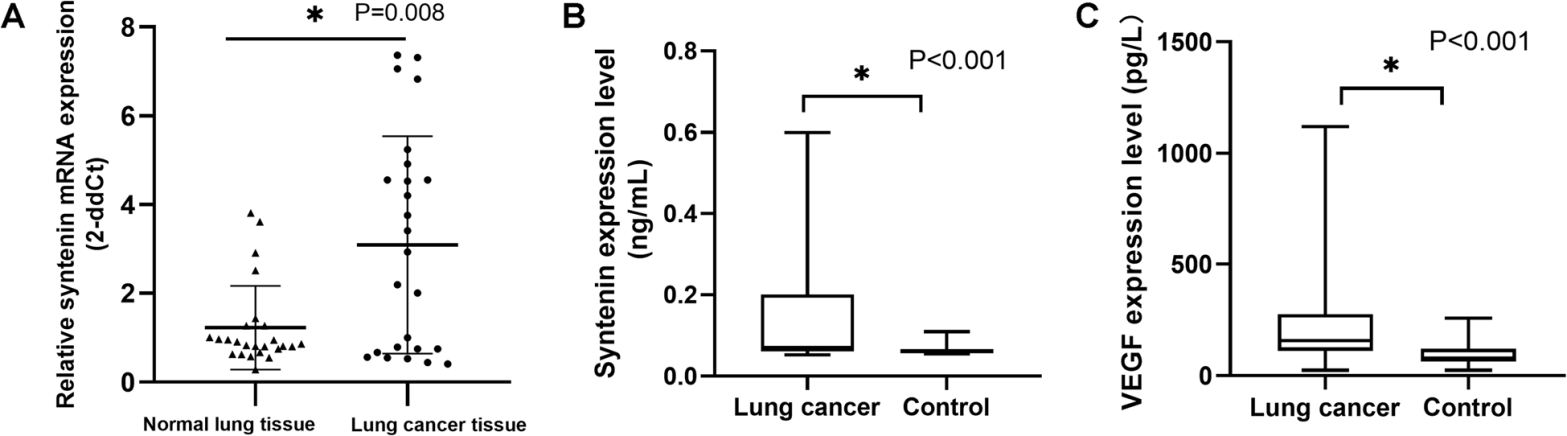
RT-qPCR analysis showed that the mRNA expression of syntenin in fresh lung cancer tissue was significantly higher than that in adjacent noncancerous tissues (*P* = 0.008) (**a**). ELISA showed that the expression level of syntenin and VEGF in serum of lung cancer patients was significantly higher than that of control group (*P* < 0.001, *P* < 0.001 respectively) (**b**, **c**)

Syntenin expression was localized mostly to the cytoplasm in syntenin-expressing cancer. Of the 191 lung cancer cases, 93 were found to have high expression of syntenin (Table 1), and 98 cases had low expression of syntenin. The overall number of lung cancer samples with high syntenin expression was 48.7%. However, all 80 cases of paracancerous normal lung tissue were low for expression of syntenin (Fig. 1a). RT-qPCR analysis showed that the expression of syntenin mRNA was significantly higher in lung cancer tissues than in paracancerous normal lung tissues (*P* = 0.008) **(**Fig. 2a). The expression of syntenin was detected in non-small cell lung cancer (NSCLC) and small cell lung cancer (SCLC); the proportion of highly-expressing samples for SCLC (57.1%, 12/21) (Fig. 1c and d) was higher than that for adenocarcinoma (51.3%, 61/119) (Fig. 1e and f) and for SSC (squamous cell carcinoma) (40.8%, 20/49) (Fig. 1g and h), whereas there was no statistically significant difference in syntenin expression among SCLC, adenocarcinoma and SSC (*P* = 0.348). There were only two cases of LCLC (large cell lung cancer) (Fig. 1b) in the study, and the expression of syntenin was low in both.

**Table 1:** Expression of Syntenin in different pathological types of lung cancer

Pathologic types	Number of sample	Positive expression (%)	Negative expression (%)	*P*
SSC	49	20 (40.8)	29 (59.2)	0.348
Adenocarcinoma	119	61 (51.3)	58 (48.7)	
LCLC^a^	2	0 (0)	2 (100)	
SCLC	21	12 (57.1)	9 (42.9)	
Total	191	93 (48.7)	98 (51.3)	

*SSC* Squamous cell carcinoma, *LCLC* large cell lung cancer, *SCLC* small cell lung cancer. ^a^: LCLC was not compared with other types of lung cancer by chi-square test

### Syntenin expression was associated with tumour size, TNM stage and distant metastasis

Among the 191 cases of lung cancer, the high expression rate for syntenin was 57.0% in 121 cases where the diameter of tumours was greater than 3 cm, while the high expression rate was 34.3% in 70 cases where the diameter of tumours was less than or equal to 3 cm, and the difference was statistically significant (*P* = 0.002). The high expression rate of syntenin in 111 cases with stage III and IV lung cancer was 55.9%, and the rate in 80 cases with stage I and II lung cancer was 38.8%, with a statistically significant difference (*P* = 0.020). Among the 58 cases of lung cancer with distant metastasis, the rate of high syntenin expression was 60.3%, while among the 133 cases of lung cancer without distant metastasis, only 58 cases were highly expressed (the rate was 43.6%), and the difference was statistically significant (*P* = 0.033). The expression of syntenin was independent of lymph node status, age, sex and performance status (*P* > 0.05) (Table 2).

**Table 2:** Syntenin expression in relation to clinical parameters and pathological characteristics

Variable	Number of sample	Syntenin		*P*
		high (%) low (%)		
Tumor size (cm)				
≤ 3	70	24 (34.3)	46 (65.7)	0.002
>3	121	69 (57.0)	52 (43.0)	
TNM stage				
I + II	80	31 (38.8)	49 (61.2)	0.020
III + IV	111	62 (55.9)	49 (44.1)	
Distant metastasis				
Negative	133	58 (43.6)	75 (56.4)	0.033
Positive	58	35 (60.3)	23 (39.7)	
Lymphnode				
metastasis				
Negative	91	38 (41.8)	53 (58.2)	0.067
Positive	100	55 (55.0)	45 (45.0)	
Sex				
Men	140	68 (48.6)	72 (51.4)	0.956
Women	51	25 (49.0)	26 (51.0)	
Age (years)				
≤ 60	84	39 (46.4)	45 (53.6)	0.579
>60	107	54 (50.5)	53 (49.5)	
Performance status				0.961
≤ 2	162	79 (50.1)	83 (53.5)	
> 2	29	14 (48.3)	15 (51.7)	

*P* value is the result of chi-square test, and the expression level of syntenin is significantly correlated with tumor size, TNM stage and distant metastasis of tumor

### Syntenin expression was associated with shorter OS and PFS

**Fig. 3:**
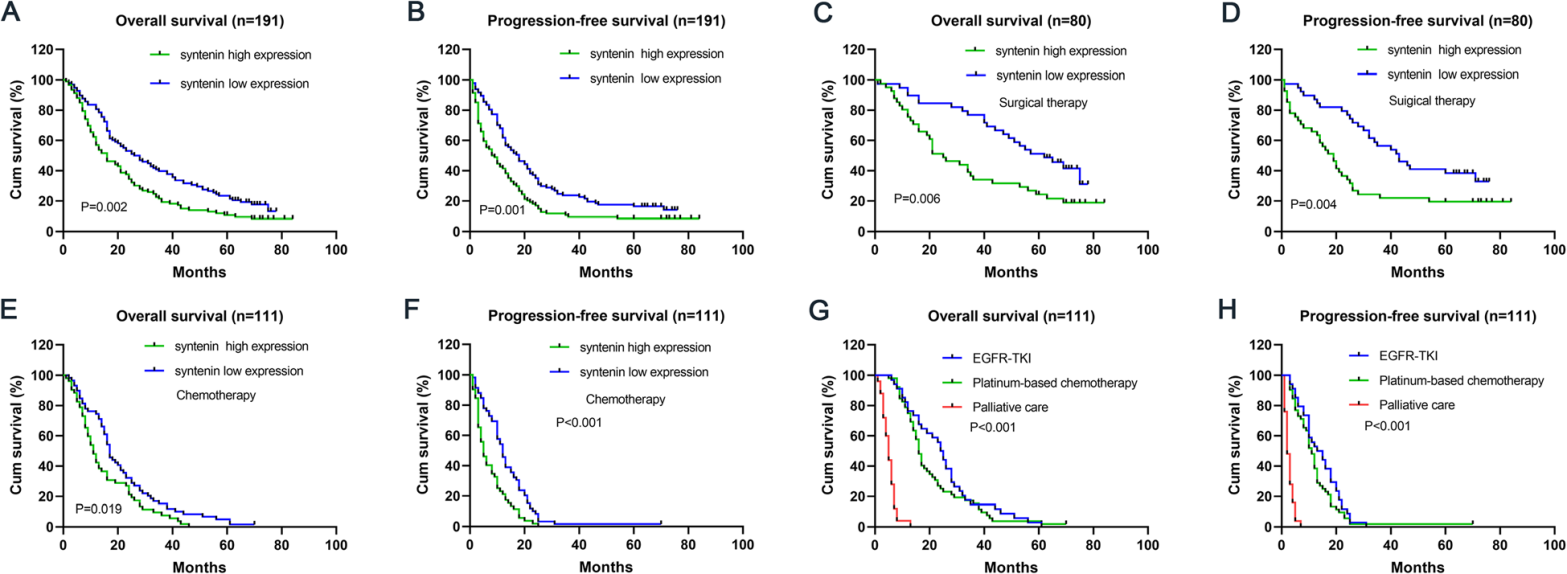
Association of syntenin expression with prognosis in patients with lung cancer. Kaplane-meier survival curves showed that OS and PFS of patients with high syntenin expression were significantly shorter than those with low syntenin expression in all cases (**a, b**). OS and PFS were significantly shorter in patients with high syntenin expression than in patients with low syntenin expression in surgical treatment (**c, d**) and chemotherapy (**e, f**). OS and PFS in EGFR-TKI treatment group were longer than those in platinum chemotherapy group and in palliative treatment group (**g** and **h**)

Kaplan-Meier survival analysis showed that the OS (*P* = 0.002) and PFS (*P* = 0.001) of the lung cancer patients with high syntenin expression were significantly shorter than those of patients with low syntenin expression, and there was a statistically significant difference between high and low groups (Fig. 3 a and b). We further analysed lung cancer patients receiving surgery and chemotherapy separately, and the results showed that the OS (*P* = 0.006) and PFS (*P* = 0.004) of lung cancer patients with high syntenin expression were significantly shorter than those with low syntenin expression in 80 surgically treated patients (Fig. 3c and d). In addition, among the 111 patients treated with chemotherapy, the OS (*P* = 0.019) and PFS *(P* < 0.001) of patients with high syntenin expression were significantly shorter than those with low syntenin expression (Fig. 3e and f). To further explore the influence of different chemotherapy treatments on the prognosis of patients, Kaplan Meier survival analysis was performed and the results showed that OS and PFS in EGFR-TKI treatment group were longer than those in platinum chemotherapy group, and OS and PFS in platinum chemotherapy group were significantly longer than those in palliative treatment group (*P* < 0.001, *P* < 0.001, respectively) (Fig. 3g and h).

### Factors affecting prognosis of lung cancer

Univariate COX proportional hazard regression analysis indicate that tumour size (*P* = 0.01 and 0.007 for OS and PFS, respectively), distant metastasis (*P* < 0.001 for OS and PFS), lymph node metastasis (*P* < 0.001 for OS and *P* PFS), performance status (*P* < 0.001 for OS and PFS) and syntenin expression (*P* = 0.004 and = 0.001 for OS and PFS, respectively) were factors predicting a poor prognosis in lung cancer patients. Unsurprisingly, surgical treatment (*P* < 0.001 for OS and PFS) indicated good prognosis in the patients. Moreover, multivariate COX proportional hazard regression analysis demonstrated that distant metastasis (*P* < 0.001 for OS and PFS), lymph node metastasis (*P* = 0.005 and = 0.004 for OS and PFS, respectively), syntenin expression (*P* = 0.006 and < 0.001 for OS and PFS, respectively) and performance status (P < 0.001 for OS and PFS) were independent risk factors for poor prognosis. In addition, we showed that surgical treatment (*P* < 0.001 for OS and PFS) was an independent protective factor for the patients, and SCLC had even worse prognosis than NSCLC (*P* = 0.047) (Tables 3 and 4). Histological types were related to OS, but not PFS, which might be due to small number of SCLC cases in the study.

**Table 3:** Univariate and multivariate COX regression analysis of prognostic factors in lung cancer patients for OS

Variable	Univariate analysis			Multivariate analysis		
	Hazard ratio	95%CI	P	Hazard ratio	95%CI	P
Tumor size	1.524	1.104~2.103	0.01	1.319	0.932~1.867	0.118
Distant metastases	4.786	3.331~6.877	< 0.001	2.893	1.916~4.370	< 0.001
Lymph node metastasis	2.102	1.537~2.877	< 0.001	1.604	1.156~2.227	0.005
Sugical treatment	0.274	0.193~0.390	< 0.001	0.489	0.320~0.749	0.001
Age	1.063	0.782~1.445	0.698			
Sex	0.796	0.559~1.135	0.207			
Syntenin expression	1.572	1.157~2.136	0.004	1.580	1.141~2.188	0.006
Performance status	8.903	5.700~13.907	< 0.001	6.371	3.750~10.822	< 0.001
Histology	0.595	0.367~0.964	0.035	0.600	0.362~0.994	0.047

**Table 4:** Univariate and multivariate COX regression analysis of prognostic factors in lung cancer patients for PFS

Variable	Univariate analysis			Multivariate analysis		
	Hazard ratio	95%CI	P	Hazard ratio	95%CI	P
Tumor size	1.557	1.130~2.144	0.007	1.162	0.830~1.627	0.383
Distant metastasis	5.754	3.888~8.515	< 0.001	2.773	1.816~4.236	< 0.001
Lymph node metastasis	2.155	1.575~2.950	< 0.001	1.613	1.165~2.234	0.004
Sugical treatment	0.245	0.169~0.353	< 0.001	0.338	0.219~0.522	< 0.001
Age	1.034	0.762~1.402	0.831			
Sex	0.767	0.540~1.091	0.140			
Syntenin expression	1.689	1.245~2.291	0.001	1.889	1.369~2.607	< 0.001
Performance status	7.578	4.778~12.020	< 0.001	4.309	2.603~7.131	< 0.001
Histology	0.640	0.395~1.035	0.069			

Distant metastasis (*P* < 0.001), lymph node metastasis (*P* = 0.001), syntenin expression

### Expression of syntenin in serum from patients with lung cancer and its relationship with VEGF expression

In the cancer group, 55 patients had NSCLC, and five patients had SCLC. The expression level of syntenin in the serum from lung cancer group was significantly higher than from the control group (*P* < 0.001) (0.071 vs 0.061 ng/mL). The VEGF level in serum from the lung cancer group was significantly higher than that from the control group (*P* < 0.001) (158.479 vs 78.612 pg/mL). Spearman correlation analysis showed that the syntenin and VEGF levels were positively correlated (correlation coefficient = 0.49, *P* < 0.001). However, further analysis revealed no significant correlation between the syntenin and VEGF levels in serum of the control subjects (correlation coefficient = 0.257, *P* = 0.171). The serum syntenin and VEGF levels in lung cancer patients and control subjects are shown in Fig. 2b and c and the correlation analysis of serum syntenin and VEGF levels in lung cancer patients and control subjects is shown in Table 5.

**Table 5:** Correlation analysis of serum syntenin and VEGF levels in lung cancer patients and control subjects

Group	Syntenin (ng/mL)	VEGF (pg/mL)	r	*P*
Lung cancer	0.071 (0.062~0.200)	158.479 (112.491~276.235)	0.49	< 0.001
Control	0.061 (0.058~0.067)	78.612 (64.824~121.209)	0.257	0.171

## Discussion

Syntenin is highly expressed in the metastatic tumour cells when compared with non-metastatic tumour cells and normal cells, and is suggested to cause metastasis by regulating the transportation of tumour cells [11, 15], resulting in poor prognosis in patients. However, the role of syntenin in the occurrence and development of lung cancer is largely unknown. Therefore, we investigated the expression of syntenin in human lung cancer tissues and serum in patients with lung cancer and the relationship between syntenin expression and clinicopathology. Our study shows that the syntenin level in the cancer tissue is significantly higher than in paracancerous lung tissue. Northern blot analysis in previous studies indicated that syntenin mRNA is abundant in adult heart and placental tissues but very low in lung tissues [25]. In addition, we found that elevated syntenin expression was correlated with tumour size, TNM stage and distant metastasis, suggesting that syntenin is involved in the occurrence and development of tumours. In breast cancer the overexpression of syntenin is found to be correlated with tumour size, lymph node status, the OS rate, and the PFS rate [15]. Kaplan-Meier analysis further suggested that the OS and PFS in high syntenin patients are significantly shorter than those in low patients. This is consistent with previous results that the abnormal expression of syntenin is related to poor clinical prognosis in glioma, breast cancer, uveal melanoma and lung adenocarcinoma [11, 15, 26, 27]. At the same time, Kaplan Meier analysis showed that the prognosis of patients treated with EGFR-TKI gefitinib is better than that of patients treated with platinum chemotherapy and palliative treatment. Previous studies show that gefitinib has significant clinical efficacy in the first-line treatment of advanced NSCLC patients [28] and the prognosis of patients with platinum chemotherapy is better than that of patients with palliative treatment. However, if the patients have high PS score, their prognosis with palliative treatment is poor. Syntenin was believed to promote the invasion and migration of various tumor cells through the integrin signaling pathway, but recent studies have shown that syntenin also promotes the invasion and migration of tumor cells without the induction of cell matrix [11, 29, 30]. Nevertheless, it is clear that elevated syntenin expression may play a vital role in the development of lung cancer.

Currently, the assessment of lung cancer prognosis relies on TNM staging. However, TNM staging is not always able to predict the prognosis of patients, and there is an urgent need for other clinical prognostic markers to assist and supplement TNM staging. Moreover, identifying novel biomarkers that predict the prognosis of lung cancer patients is helpful for the selection of treatment regimens and the improvement of survival rate. This study showed that the overexpression of syntenin in lung cancer tissues is correlated with poor prognosis in lung cancer. Univariate and multivariable COX proportional hazard regression analyses further showed that the overexpression syntenin is an independent risk factor for lung cancer patients with poor prognosis. Therefore, syntenin may be used as a new prognostic marker for lung cancer.

At present, VEGF has been widely recognized to play an indispensable role in angiogenesis during tumour growth [31], and VEGF levels in serum from lung cancer patients have also been measured in previous studies. However, there is no studies that report the level of syntenin in serum from lung cancer patients. In this study, we found that the serum level of syntenin in lung cancer patients is significantly higher than that in control subjects, which may be related to the high tumor burden of lung cancer patients. We also confirmed that the level of serum VEGF is higher from lung cancer patients than from control subjects as found previously for NSCLC [32]. In addition, we further found that there is a positive correlation between serum syntenin and VEGF levels in patients with lung cancer, suggesting that the increased expression of VEGF may be related to the overexpression of syntenin. Since the effect of syntenin on VEGF downstream pathway may be different in different pathological types of lung cancer, our study did not further investigate how syntenin mediates the increase of VEGF expression in lung cancer patients. With the gradual increase of tumor volume, hypoxia appears, resulting in increased expression of hypoxia-inducible factor-1 α (HIF-1 α) and VEGF to promote angiogenesis in tumor. It has been shown that the overexpression of HIF-1 α in lung cancer can regulate VEGF transcription and neovascularization in a positive feedback way. Therefore, the increase of HIF-1 α reflects the occurrence and progress of cancer [33]. HIF-1 α and VEGF overexpressed in lung cancer are secreted into the blood and can be used to diagnose the cancer [34], Syntenin is shown to inhibit apoptosis [35] and inhibition of syntenin will reduce the activity of VEGF [19]. In melanoma, syntenin induces angiogenesis by activating Akt, leading to the expression of HIF-1α and the transcription of IGF-binding protein-2 (IGFBP-2), which induces the production of VEGF in endothelial cells and angiogenesis [36]. In addition, the inhibition of syntenin protein expression reduces microvascular branching in vivo and the number of tumour vessels in an orthotopic xenograft mouse model [11]. Studies have shown that syntenin is abnormally expressed in pulmonary and hepatic veins during mouse embryonic development, indicating that it plays an important role in regulating the function of endothelial cells [37]. All of the above studies indicate that syntenin can promote angiogenesis in tumour through different molecular pathways.

This study was a combination of retrospective and prospective studies. The patients experienced different anticancer treatments and had different levels of tumour progression, adverse reactions to anticancer treatment, and nutritional statuses; all of these factors have not been taken into account. The entire study population was from the Haikou Hospital, and almost all the patients were from Hainan Province, which had certain bias and limitations.

## Conclusion

Our work has demonstrated that syntenin expression is increased in lung cancer tissues and serum of lung cancer patients, and overexpression of syntenin is significantly correlated with poor prognosis of lung cancer patients. Combined with previous studies, it is clear that syntenin plays an important role in regulating metastasis and angiogenesis in lung cancer. Therefore, syntenin is expected to become a new diagnostic and prognostic marker for lung cancer, and syntenin-targeted therapy is expected to be a new supplement to traditional treatment methods. Due to the limitations described above, further study is needed to investigate the role of syntenin in lung cancer at molecular and cellular levels to develop targeted therapy.

## Data Availability

The datasets used during the current study are available from the corresponding author on reasonable request.

## Abbreviations

A: Absorbance
COPD: Chronic obstructive pulmonary disease
ELISA: Enzyme-linked immunosorbent assay
IRS: Immunoreactive score
LCLC: Large cell lung cancer
MDA-9: Melanoma differentiation-associated gene-9 (MDA-9)
NSCLC: Non-small cell lung cancer
OS: Overall survival
PFS: Progression-free survival
PP: Percentage of positive cells
PS: Performance status
RT-qPCR: Quantitative real-time polymerase chain reaction
SCLC: Small cell lung cancer
SI: Staining intensity
SSC: Squamous cell carcinoma
VEGF: Vascular endothelial growth factor
VEGFR: Vascular endothelial growth factor receptor
